# Post award arbitral tribunal's mandate under the UNCITRAL Model Law and national laws based thereon

**DOI:** 10.1016/j.heliyon.2021.e07556

**Published:** 2021-07-13

**Authors:** Omar Hisham Al Hyari, Abdullah Rabee Al Ani

**Affiliations:** British University in Dubai, Dubai, United Arab Emirates

**Keywords:** Arbitration, UNCITRAL Model Law, Model Law jurisdictions, Arbitrators' mandate, Award, Correction, Interpretation, Additional award

## Abstract

The UNCITRAL Model Law on International Commercial Arbitration provides for the extension of the mandate of the arbitral tribunal post issuance of the final award for the issuance of correction, interpretation, additional award, and remittance of the award back to the arbitral tribunal to remove grounds for challenging the award. Using a doctrinal approach, this paper examines the deviations of the national laws of adopting jurisdictions from the Model Law in regards to this extended mandate, and evaluates the improvements and drawbacks in these deviations. Mainly, the findings of this paper are that, of the many deviations, the positive changes are those that provide comfortable and lenient default provisions for the benefit of inexperienced parties, and since correction, interpretation, additional award, and remittance are useful provisions that are designed to help self-rectify the arbitral process, without adversely delaying it, then the changes that increase the efficacy of these provisions are welcomed. On the other hand, unnecessary deviations are seen as drawbacks that hinder the harmonization of national arbitration laws aimed at by the Model Law. The adopting jurisdictions shall be limited to those acknowledged as such by the UNCITRAL.

## 1 Introduction

The UNCITRAL Model Law on International Commercial Arbitration was enacted by the United Nations Commission on International Trade Law (UNCITRAL, 1985) in 1985^1^ to provide an arbitration law format that harmonizes national arbitration laws and aligns these with the 1958 New York Convention on the Recognition and Enforcement of Foreign Arbitral Awards. According to the UNCITRAL, 116 jurisdictions in 83 States have adopted the Model Law as of the time of issuance of this paper.^2^

The Model Law provides that the mandate of the arbitral tribunal terminates upon the issuance of the final award,^3^ with the exception of article 33 and sub-article 34.4 which provide for correction, interpretation, issuance of an additional award, and remittal of the award back to the arbitral tribunal for the sake of removing grounds for challenge against the award. The importance of these provisions lies in that they allow the arbitral tribunal to correct mistakes and dispel uncertainty in its award, when required, so as to make sense of and give proper effect to the award, and ultimately, the arbitral process can self-rectify itself. This paper shall identify and evaluate the deviations in article 33 and sub-article 34.4 between the Model Law and the national arbitration laws based thereon.^4^ The significance of this topic is in understanding what these deviations are, and analyzing whether they are improvements or defective modifications to the Model Law; this topic was chosen as there seems to be no such comparative study in the literature. In addition to the introduction and methodology sections, the paper's structure comprises of two sections followed by a conclusion; these two sections shall be as follows:

• Section 1 - Correction and interpretation of the award and the making of an additional award; this section shall firstly deal with the unique aspects of each provision separately, and then deal with the common matters jointly.
• Section 2 - Remittance of the Award.

## 2 Methodology

This paper's research is doctrinal and includes the review of the 116 adopting jurisdictions' arbitration laws, in addition to the available literature and case law on article 33 and sub-article 34.4.

## 3 Correction and interpretation of the award and the making of an additional award

The Model Law allows, under article 33, the arbitral tribunal to correct and interpret its award and issue an additional award subsequent to the issuance of the final award. Hereunder, each of these powers of the arbitral tribunal shall be looked at individually and matters in common between the powers shall be considered together under the sub-heading ‘General Considerations’.

### 3.1 Correction

The Model Law enables a party to request the arbitral tribunal to correct its award. Such a correction is not intended to re-open a case,^5^ but to prevent certain errors from misrepresenting the intentions of the arbitral tribunal and/or invalidating the award. Sub-article 33.1.a of the Model Law specifies that a correction is applicable to ‘any errors in computation, any clerical or typographical errors or any errors of similar nature’.^6^ ^7^ ^8^ However, some of the adopting jurisdictions have not followed this phrasing.^9^ For example, the Egyptian Arbitration Law states that correction is applicable to what befalls in the award of material mistakes whether clerical or arithmetic.^10^ This aberration may have the effect of altering the scope of correction which has the general drawback of de-harmonizing the arbitration laws of the adopting jurisdictions with respect to correction, a matter that defeats one of the purposes of the Model Law.

While the Model Law does not allow the parties to agree to prevent the submission of a correction request, 11 some adopting jurisdictions have permitted them to do so.^12^ The drawback of agreeing out of correction is that an award may be defective due to a mistake that would otherwise be correctable, this would unnecessarily risk invalidating the award especially for inexperienced parties that may unwittingly agree to opt out of correction.

Another mandatory characteristic of correction under the Model Law is the ability of the arbitral tribunal to correct its award on its own initiative. The tribunal must issue such a correction within 30 days from the date of issuance of the award. However, unlike for correction at the request of a party, the duration for the issuance of a correction on the tribunal's own initiative may not be extended.^13^ Furthermore, it is implied by the fact that correction is issued by the tribunal on its own initiative that the tribunal is not obliged to afford an opportunity to the parties to make submissions, to oppose or otherwise direct the tribunal's decision, on such correction.^14^ While most adopting jurisdictions do provide for self-correction, some jurisdictions have made changes in the details of this provision. Certain adopting jurisdictions provide for a different time limit for the issuance of the self-correction,^15^ while other adopting jurisdictions allow for extension of the time limit - albeit by a limited duration.^16^ It is submitted that while the time limit for self-correction set under the Model Law is reasonable, as evidenced by the fact that most adopting jurisdictions have retained the same time limit, minor deviations in the duration of the time limit by some adopting jurisdictions should not have a significant impact on the utility of this provision. However, adopting jurisdictions that make drastic changes to the same, such as Qatar, which sets this time limit at 7 days, reduce the effectiveness of this provision. On the other hand, adding the ability to extend this time limit by a limited duration can be a positive step, in that it can give the arbitral tribunal more leeway to make its correction when this is really needed. In contrast, there are adopting jurisdictions that have not stated a time limit for the issuance of self-correction.^17^ This lack of specificity is undesirable as it will cause uncertainty. Finally, at least one adopting jurisdiction has removed self-correction altogether.^18^ This deviation is a drawback since the arbitral tribunal is at the best position to identify an error in need of correction, it could identify mistakes better/faster than the parties would.

### 3.2 Interpretation

The Model Law allows a party to request the arbitral tribunal to interpret its award. Accordingly, if there is ambiguity in the award, a party may request the tribunal to issue a clarification.^19^ Sub-article 33.1 of the Model Law states that interpretation must be requested for a specific ‘point or part of the award’,^20^ and thus the requesting party must indicate where the interpretation is required, in its request, and must convince the arbitral tribunal of the ambiguity therein. Furthermore, since the Model Law does not limit the possibility of interpretation to a specific part of the award, it is submitted that interpretation may be used to remove ambiguity in any part including the reasoning or the dispositive portions of the award.^21^ In comparison, some adopting jurisdictions do specify the portions of the award that may be interpreted, but limit the same to the dispositive portions of the award.^22^ This limitation may reduce the usefulness of this provision as there could be an ambiguity in another part of the award that does not cast doubts on its dispositive part. While it may be argued that the limitation should improve the swiftness of arbitrations, by reducing the number of interpretation requests, this could be at the expense of upholding the fairness of the award.

The provision for interpretation under the Model Law is only applicable on the explicit agreement of the parties. Some adopting jurisdictions provide for interpretation by default i.e. unless otherwise agreed by the parties,^23^ and others make interpretation a mandatory provision.^24^ In contrast, some adopting jurisdictions do not allow for interpretation at all.^25^ Derains and Schwartz note that interpretation is infrequently requested for and even less so assented to in international arbitration,^26^ and thus the alteration of its availability and non-mandatory characteristic may not be as significant as it may be thought. Be that as it may, since a party that is less experienced in arbitration may not pay attention to details of the arbitral process at the time of contracting, and once a dispute arises, it usually becomes difficult for parties to agree on any matter, and considering that an ambiguous award may be difficult to enforce, especially if the ambiguity is in its dispositive part, it would appear that the interpretation provision should be mandatory.

Finally, while self-issuance of interpretation is unavailable under the Model Law, a small number of adopting jurisdictions do allow for this.^27^ This is a positive deviation from the Model Law as it creates consistency with the correction provision, and it does not cause any unnecessary delay to the arbitral process since the arbitral tribunal cannot be expected to take this step unless it is necessary.

### 3.3 Additional award

The Model Law allows a party to apply to the arbitral tribunal to issue an additional award subsequent to the issuance of the final award. Sub-article 33.3 of the Model Law stipulates that an additional award may only be issued towards a claim, or counterclaim, that had been submitted during the arbitral process, but inadvertently omitted from the final award.^28^ ^29^ Unlike correction and interpretation, an additional award may require the arbitral tribunal to re-open the case, in relation to the inadvertently omitted claim, so as to fully dispense with the dispute under arbitration.^30^ However, one adopting jurisdiction^31^ provides for amendment of the final award, instead of issuance of an additional award; stating that the arbitral tribunal may ‘amend the award so as to correct an injustice caused by an oversight on the part of the arbitral tribunal’. Furthermore, a few adopting jurisdictions allow for the review of cost awarded, in case material information has come to light after the issuance of the final award.^32^ Finally, at least one adopting jurisdiction^33^ provides for the award of arbitration cost, in addition to the Model Law's additional award provision, if the same had not been issued in the final award. These are unnecessary deviations that contradict the harmonization aimed at by the Model Law. As evident by the fact that the vast majority of adopting jurisdictions provide for an additional award rather than an amendment, it is submitted that the former is the internationally accepted form for ruling on omitted claims. This is in addition to the fact that the existence of two awards, i.e. amended and amending awards, with different dipositive parts may create practical enforcement and/or nullification-related problems. The review of costs upon discovery of ‘material information’ is a vague deviation. If intended to cover the case of discovery of new evidence, then this is a case that most arbitration laws do not consider as a sufficient reason for extending the tribunal's mandate for different reasons including that the tribunal should not be blamed for the emergence of fresh evidence. Arbitration costs are nearly always included in the relief sought by each party. They should, therefore, be decided upon in the final award. If this is inadvertently not done, an additional award can be requested.

While under the Model Law, parties may agree out of additional awards, some adopting jurisdictions have made it a mandatory provision,^34^ and others have removed the provision entirely.^35^ While allowing freedom – under the Model Law - to agree on the availability of an additional award is appreciated, parties that lack sufficient knowledge in arbitration may not pay attention to the importance of this provision at the time of contracting, and so it could be argued that the former deviation is reasonable as it ensures that this critical provision is available for those less experienced parties. As for the latter deviation, it is certainly a major drawback in that it reduces the efficacy of the arbitral process and its ability to fully dispense with all matters under dispute.

Finally, as with the self-issuance of interpretations above, some adopting jurisdictions also allow for the self-issuance of additional awards,^36^ which is also unavailable under the Model Law. This is a positive addition as it creates consistency with the correction provision, and it does not cause any unnecessary delay to the arbitral process since the arbitral tribunal cannot be expected to take this step unless it is necessary.

### 3.4 General considerations

The Model Law prescribes a time limit for a party to apply to the arbitral tribunal for an article 33 request; this is 30 days from the date of receipt of the arbitral award by the requesting party,^37^ and the parties are granted the freedom to adjust this time limit by agreement. Some adopting jurisdictions have altered the default time limit^38^ and/or the ability to adjust the time limit by agreement.^39^ While changing the default time limit for an article 33 request may not have impact for experienced parties, as the parties may change this by agreement, it may have an adverse impact on less experienced parties, especially when the default time limit is drastically shortened - for example, under the Qatari Arbitration Law, the default time limit is 7 days. Furthermore, changing the ability to agree on these time limits restricts the principle of autonomy of the parties and the flexibility of the arbitral process to cater to individual cases as required. Finally, some adopting jurisdictions allow a party to make an article 33 request after the expiry of the time limit for a request, upon the approval of the arbitral tribunal^40^ or the court;^41^ this seems to be a positive deviation for those exceptional circumstances where this would be justified – albeit having the arbitral tribunal decide on extension of the time limit is more appealing as it maintains the arbitration's autonomy from the court.

Similarly, the Model Law prescribes time limits on the arbitral tribunal for the issuance of article 33 dispositions; these are 30 days for the issuance of corrections and interpretations and 60 days for the issuance of additional awards – all from the date of receipt of the request by the arbitral tribunal.^42^ However, unlike for the time limit of a request, the Model law does allow the arbitral tribunal to extend the time limits for the issuance of an article 33 disposition as it sees fit. Some adopting jurisdictions have altered either the default time limits of issuance,^43^ the ability of the arbitral tribunal to extend these time limits,^44^ or have set maximum durations of extension of these time limits by the arbitral tribunal.^45^ While changing the default time limits may not have significant impact, as the arbitral tribunal is allowed to extend these, the time limits set by the Model Law are reasonable, as evidenced by the conformity to the same by the majority of the adopting jurisdictions, and deviation by a few jurisdictions does not seem justifiable and is inconsistent with the desired harmonization aimed at by the Model Law. On the other hand, disallowing or limiting the ability of the arbitral tribunal to extend these durations may lead the process by which an article 33 disposition is decided and issued to be inequitable especially in additional awards.

The Model Law stipulates that the non-requesting party be notified, by the requester, of an article 33 request, at the same time the request is submitted to the arbitral tribunal.^46^ However, in case an arbitral tribunal decides to issue a correction on its own initiative, the Model Law does not specify that the parties be notified of the tribunal's decision before making the disposition. Some adopting jurisdictions further specify that the arbitral tribunal must give opportunity to the non-requesting party to present its case, before making an article 33 disposition.^47^ This deviation is supposed to have no impact as the same requirement is already implied under the Model Law.^48^ However, the added clarity is welcomed. In contrast, a few adopting jurisdictions make no mention of the requirement for notification.^49^ Finally, some adopting jurisdictions specify that the arbitral tribunal must first notify the parties before issuing a self-correction.^50^ The requirement for notification to the non-requesting party is essential to ensure equity of an article 33 process/disposition, and so, removing this requirement is a negative deviation. In contrast, adding the requirement for notification of a correction on the arbitral tribunal's own initiative is an improvement to the Model Law that would ensure a higher level of fairness to a self-correction, at virtually no drawback, or at the very least, the parties would be more agreeable to a self-correction, if they had been notified earlier of the arbitral tribunal's decision to issue the same.

While the Model Law does not permit the court to issue an article 33 disposition, a few adopting jurisdictions do. For example, under the UAE Arbitration Law,^51^ if the arbitral tribunal does not dispense with an article 33 request in accordance with the provisions of the law, then the concerned party can ask the court to issue a disposition on the request. Another deviation that some adopting jurisdictions have made is to allow the court the authority to decide on an article 33 request only if the re-assembly of the original arbitral tribunal, for the sake of deciding on the request, is not possible.^52^ Unlike the former deviation, the latter one may be acceptable given that it is very limited and is expected to be applied rarely. However, both deviations contradict the fact that the parties have agreed to arbitrate rather than to litigate in the first place.

## 4 Remittance of the award

Under the Model Law,^53^ an arbitral award may be remitted back to the arbitral tribunal, during a setting aside action, to remove the grounds challenging the award.^54^ It is implied under the Model Law that the parties cannot contract out of remission.^55^ While most adopting jurisdictions allow for remission, some do not provide for remission at all,^56^ and others have made remission non-mandatory.^57^ As remission can be considered the last line of defense against the setting aside of an award, then removing this provision would be a drawback in that it would increase the cases an award is set aside - thereby reducing the ability of the arbitral process to rectify itself. Furthermore, changing the mandatory characteristic of remission may have an adverse effect on parties unfamiliar with arbitration, in that they may unwittingly agree to its exclusion although it can in fact save the effort spent on the arbitral process by avoiding the setting aside of an award wherever possible.

A party must request the court to order remission under the Model Law. However, some adopting jurisdictions allow the court to remit an award without the request of a party.^58^ Since the court is best suited to judge whether a ground for setting aside the award may be rectified by remission, then it is contended that it should be able to order remission on its own initiative. This would be especially beneficial for parties unfamiliar with arbitration that may otherwise miss their opportunity to make such a request to the court. Hence, it is argued that this alteration should be adopted by the Model Law as it would reduce the number of successful cases of setting aside of an arbitral award, and thereby increase the efficacy of the arbitral process.

The Model Law does not specify a timeframe by which the outcome of remission must be obtained, nor by how long the setting aside procedure may be suspended for remission. Accordingly, this period is open to the court to decide. However, some adopting jurisdictions do specify such a timeframe.^59^ It is submitted that specifying a timeframe by law may encourage a hastier remittal process especially in adopting jurisdictions where the laws have imposed very short periods that would not suit all cases. The Model Law provides the better alternative to leave the timeframe to the court to assess based on the circumstances of each case.

Under the Model Law, an award may be remitted back to the arbitral tribunal ‘in order to give the arbitral tribunal an opportunity to resume the arbitral proceedings or to take such other action as in the arbitral tribunal's opinion will eliminate the grounds for setting aside’. Although this phrasing does not clarify whether the content of the award may be changed,^60^ it is implied that this is allowed. However, the UAE Arbitration Law provides that the outcome of remittance shall not affect the content of the award.^61^ This deviation would reduce the efficacy of the arbitral process as it would reduce the number of cases where remission could save an award from annulment.

## 5 Conclusion

This paper aimed to bridge a gap in the literature in regard to the difference in the arbitral tribunal's post final award mandate between the Model Law and the national laws based thereon. Unlike what may be thought, the tribunal's mandate does not wholly end by issuing the final award, and in fact the arbitral tribunal may issue a correction, interpretation or additional award. Furthermore, the arbitral tribunal may even change the final award upon remittance of the same by the court. The importance of this topic lies in the key feature of the post award powers that allow the arbitral tribunal to rectify itself, and the fact that these powers are inconsistent between the Model Law adopting jurisdictions. Hence, this comparison between the model law and adopting jurisdictions is useful to explore rooms for improvement to the model law and identify unnecessary deviations that defeat the harmonization aimed at by the Model Law. While some of the changes referred to in this paper are minor - whether this is due to the changes being nominal,^62^ or of little practical significance^63^ - others are of considerable effect.^64^ Furthermore, while some of the deviations reflect a jurisdiction's priority to the outcome of arbitration, over the preferences of the drafters of the Model Law - such as prioritizing the swiftness of the arbitral process and finality of the award over the equity and clarity of the award - some changes are perhaps not well thought out and render the provisions of article 33 and sub-article 34.4 less effective.^65^ However, the authors are of the opinion that default provisions, such as the time limit for raising an article 33 request, which may be changed by the agreement of the parties, should be made in such a way as to assume that the parties to the arbitration are inexperienced, so as not to prejudice those with less understanding of these provisions. Furthermore, as the provisions of article 33 and 34.4 improve the efficacy and autonomy of the arbitral process, the deviations that increase the scope and accessibility of these provisions are welcomed.^66^ Finally, small deviations, which are seemingly unjustified, should be removed to improve the harmony between arbitration laws aimed for by the Model Law.

Some of the more prominent positive deviations include adding the ability of the limited extension of the timeframe for the issuance of self-correction, adding the provision for self-interpretation and self-issuance of additional awards, making interpretation and additional awards mandatory, and allowing the court to remit an award to the arbitral tribunal without the request of a party. On the other hand, some of the more prominent negative deviations include changing the mandatory characteristic of correction, removing the provision for self-correction, limiting the scope of interpretation to the dispositive portion of the award, removing the provision for interpretation altogether, removing the provision for additional award altogether, disallowing the parties from agreeing on the time limit for an article 33 request, disallowing or limiting the ability of the arbitral tribunal to extend the time limit for the issuance of an article 33 disposition, allowing the court to issue an article 33 disposition in case the arbitral tribunal fails to do so, removing the provision for remittance altogether, changing the mandatory characteristic of remittance, specifying a timeframe for the arbitral tribunal to carry out the re-consideration/arbitration after an award is remitted, and limiting the scope of the outcome of a remission and specifying that it shall not affect the outcome of the award.

## Declarations

### Author contribution statement

Omar Hisham Al Hyari, Abdullah Rabee Al Ani: Conceived and designed the experiments; Performed the experiments; Analyzed and interpreted the data; Contributed reagents, materials, analysis tools or data; Wrote the paper.

### Funding statement

This research did not receive any specific grant from funding agencies in the public, commercial, or not-for-profit sectors.

### Data availability statement

Data included in article.

### Declaration of interests statement

The authors declare no conflict of interest.

### Additional information

No additional information is available for this paper.

## Footnotes

1 And amended in 2006. Hereafter referred to as ‘the Model Law’.

2 The list of adopting jurisdictions is provided on the UNCITRAL website: https://uncitral.un.org.

3 The Model Law, under article 32, states that the tribunal's mandate shall terminate upon the termination of the arbitral proceedings, and that the arbitral proceedings shall terminate upon, *inter alia,* the issuance of the final award.

4 As the Model Law is concerned with international arbitration, the scope of this paper shall exclude the legislation related to domestic arbitration, wherever there is a difference between domestic and international arbitration.

5 During the drafting process of the Model Law, it was unsuccessfully argued that correction and interpretation should not be included because it might have the effect of re-opening the case (UNCITRAL, Travaux préparatoires of the UNCITRAL's Model Law on International Commercial Arbitration – document title: A/CN.9/246, annex; A/CN.9/263 and Add.1–2, A/CN.9/264 (329th meeting; dated 18th June 1985), link: https://uncitral.un.org/sites/uncitral.un.org/files/media-documents/uncitral/en/329meeting-e.pdf. It is internationally accepted that correction shall not have this effect, see Gaillard et al. (1999), Goldman on International Commercial Arbitration” (The Hague, Boston, London: Kluwer Law International, 1999), referred to by Williams and Buchanan (2001), “Correction and interpretation of awards under article 33 of the Model Law”, 2001, 4(4), Int. A.L.R, 121–122.

6 It has been held that the phrase “errors in computation” includes mistakes in calculation, the use of incorrect data in calculations, the omission of data in calculations by the tribunal, and that an arbitral tribunal is not empowered under this article to correct errors of judgment, whether of law or of fact; see CLOUT case No. 208 (Vanol Far East Marketing Pte. Ltd. v. Hin Leong Trading Pte. Ltd., High Court, Singapore, 27 May 1996) – link to CLOUT case: https://www.uncitral.org/clout/clout/data/sgp/clout_case_208_leg-1432.html. See also CLOUT case No. 267 (Zimbabwe Electricity Supply Commission v. Genius Joel Maposa, Harare High Court, Zimbabwe, 29 March and 9 December 1998) where the arbitral tribunal had made a factual error in the calculation of the back-pay to which one of the parties was entitled, and the Court found that the error made by the arbitral tribunal was one of computation within the meaning of article 33 – link to CLOUT case: https://www.uncitral.org/clout/clout/data/zwe/clout_case_267_leg-1490.html. Furthermore, corrections that would recall, reverse or otherwise change the meaning of the award do not fall within the scope of article 33, see Oberlandesgericht Stuttgart, Germany, 1 Sch 13/01, 20 December 2001 (an arbitral award which revised an earlier final award was set aside), available at http://www.dis-arb.de/en/47/datenbanken/rspr/olg-stuttgart-case-no-1-sch-13-01-date-2001-12-20- id160; Oberlandesgericht Dresden, Germany, 11 Sch 01/00, 11 December 2000, available at http://www.dis-arb.de/en/47/datenbanken/rspr/olg-dresden-case-no-11-schh-01-00-date-2000-12-11-id66. See also Tan Poh Leng Stanley v. Tang Boon Jek Jeffrey, High Court, Singapore, 30 November 2000 [2000] SGHC 260 [2000] 3 SLR(R) 847, where an arbitral award which revised an earlier final award was set aside, and the decision of the High Court was reversed on appeal (Tang Boon Jek Jeffrey v. Tan Poh Leng Stanley, Court of Appeal, Singapore, 22 June 2001 [2001] 3 SLR 237) but the appeal turned on the definition of when there is a final award and did not refute the principal ruling that the arbitral tribunal is not empowered to recall or revise a final award, UNCITRAL 2012 Digest of Case Law on the UNCITRAL Model Law on International Commercial Arbitration (United Nations Pubns 2012), at page 82 – link: https://www.uncitral.org/pdf/english/clout/MAL-digest-2012-e.pdf.

7 It has been judicially confirmed that the phrase “clerical or typographical errors” includes mistakes made in the course of typing or drafting the arbitral award, see CLOUT case No. 625 (Relais Nordik Inc. v. Secunda Marine Services Limited and Anor, Federal Court, Canada, 12 April 1990) – link to CLOUT case: https://www.uncitral.org/clout/clout/data/can/clout_case_625_leg-1866.html.

8 The phrase “any errors of similar nature” has sometimes been widely interpreted to include mistakes made by the parties. For instance, a Singapore tribunal has found that correction is applicable where one of the parties, by mistake, forgot to include certain expenses in its bill of costs upon which the final award on costs was made, see CLOUT case No. 207 (Arb. No. 6 of 1996, Singapore International Arbitration Center, 6 February 1998, anonymous parties, unpublished) – link to CLOUT case: https://www.uncitral.org/clout/clout/data/fra/clout_case_207_leg-1430.html.

9 Such as the arbitration laws of Belarus, Bulgaria, Connecticut (USA), Egypt, Estonia, Florida (USA), Illinois (USA), Iran, Maldives, Newfoundland and Labrador (Canada), Northwest Territories (Canada), Norway, Nova Scotia (Canada), Nunavut (Canada), Oman, Poland, Prince Edward Island (Canada), Qatar, Saudi Arabia, Serbia, Slovakia, Texas (USA), Tunisia, UAE, and Zambia.

10 It should be noted that all translations from native texts provided in this paper are not obtained from official sources.

11 This was the intention of the group that was responsible for the drafting of the Model Law (referred to by the UNCITRAL and hereafter as ‘the Working Group’), see Holtzmann and Neuhaus (1989), “A Guide to the UNCITRAL Model Law on International Commercial Arbitration: Legislative History and Commentary” (Netherlands: Kluwer Law and Taxation Publishers, 1989), 889–907.

12 Such as the arbitration laws of Belarus, Prince Edward Island (Canada), Newfoundland and Labrador (Canada), Northwest Territories (Canada), Nova Scotia (Canada), Nunavut (Canada), Qatar, and Zambia.

13 As noted by David and Amy, neither the 30-day time limit for issuing a self-correction, nor the inability of the arbitral tribunal to extend that time limit, may be changed by the agreement of the parties, Williams and Buchanan (2001), “Correction and interpretation of awards under article 33 of the Model Law”, 2001, 4(4), Int. A.L.R, 121–122.

14 This is also implied by the fact that the non-extendable 30-day time limit for issuing a correction may not be sufficient for allowing submissions by the parties.

15 Such as Austria (28 days), Bangladesh (14 days), Bhutan (20 days), Bulgaria (60 days), Liechtenstein (28 days), Paraguay (15 days), Qatar (7 days), Saudi Arabia (15 days), Scotland (28 days), Slovakia (60 days), and Sri Lanka (14 days).

16 Such as the Egypt (up to 30 days), Oman (up to 30 days), and UAE (up to 15 days).

17 Such as Connecticut (USA), Estonia, Florida (USA), Germany, Illinois (USA), Newfoundland and Labrador (Canada), Northwest Territories (Canada), Nova Scotia (Canada), Nunavut (Canada), Prince Edward Island (Canada), Texas (USA), and Zambia.

18 See the law of Serbia.

19 Similar to correction, an interpretation is not meant to re-open the case, but to clarify the intent of the arbitral tribunal to ensure that the award is enforced in line with its intent.

20 The Working Group stated that this requirement was included to avoid abusing the provision. See Holtzmann and Neuhaus, 1989, “A Guide to the UNCITRAL Model Law on International Commercial Arbitration: Legislative History and Commentary” (Netherlands: Kluwer Law and Taxation Publishers, 1989), 889–907.

21 During discussions, at the time of drafting the Model Law, between the Working Group and national delegates, there was an attempt by some delegates to reduce the scope of interpretation to only the reasons of the award, but the working group decided that it should also be applicable to the dispositive part of the award (arguing that if the dispositive portions of the award were subject to some ambiguity, it might be difficult to enforce or implement the award); see Holtzmann and Neuhaus (1989), “A Guide to the UNCITRAL Model Law on International Commercial Arbitration: Legislative History and Commentary” (Netherlands: Kluwer Law and Taxation Publishers, 1989), 889–907. Furthermore, interpretation cannot be used to change the dispositive part or add to the reasons of the award. See Baker and Davis, 1992. The UNCITRAL Arbitration Rules in Practice (Kluwer Law and Taxation Publishers 1992), referenced to by Williams and Buchanan (2001), “Correction and interpretation of awards under article 33 of the Model Law”, 2001, 4(4), Int. A.L.R, 121–122.

22 Such as Egypt, Oman, Jordan, Saudi Arabia, and UAE.

23 Such as Belarus, Qatar, and UAE.

24 Such as Bangladesh, Bhutan, Bulgaria, Cambodia, Connecticut (USA), Egypt, Estonia, Florida (USA), Germany, Illinois (USA), Iran, Jordan, Kenya, Lithuania, Maldives, Malta, Nigeria, Oman, Poland, Saudi Arabia, Scotland, Serbia, Slovakia, Spain, Texas (USA) Tunisia, and Turkey.

25 Such as Denmark, Norway, and Zambia.

26 Derains and Schwartz (1998) Guide to the New ICC Rules of Arbitration (Kluwer Law International 1998), referred to in Williams and Buchanan (2001), “Correction and interpretation of awards under article 33 of the Model Law”, 2001, 4(4), Int. A.L.R, 121–122.

27 These are specifically Scotland, Iran, and Lithuania.

28 There is a difference between a claim that had been omitted from an award, and a party's submission in relation to a claim that had not been referred to in the award; only the former would warrant an additional award. Fouchard rightly states that it is an internationally accepted principle in arbitration that the arbitral tribunal is ‘not required to address every factual or legal allegation submitted by the parties, let alone every argument put forward’ - Emmanuel Gaillard et al., 1999, Goldman on International Commercial Arbitration” (The Hague, Boston, London: Kluwer Law International, 1999), referenced to by Hill (2018), 'Claims that an Arbitral Tribunal Failed to Deal With an Issue: The Setting Aside of Awards under the Arbitration Act 1996 and the UNCITRAL Model Law on International Commercial Arbitration' (2018) 34 Arbitration International, 8.

29 The assumption that an arbitral tribunal had failed to deal with a claim (or part thereof), does not necessarily justify an additional award, as it can be understood that the arbitral tribunal chose not to explicitly deal with that claim due to its irrelevance. For example, by dealing with one claim or issue, the tribunal may have dispensed with another claim or issue. It is submitted that, although the arbitral tribunal must deal with all submitted claims, the award does not necessarily have to specify how the tribunal disposed of the same (if it could be inferred that the arbitral tribunal has dealt with an allegedly omitted claim, then an additional award may not be used). See Williams and Buchanan, 2001, “Correction and interpretation of awards under article 33 of the Model Law”, 2001, 4(4), Int. A.L.R, 121–122. See Singapore case TMM Division Maritima SA de CV v Pacific Richfield Marine Pte Ltd [2013] SGHC 186 at [77], where the court confirmed that an issue may be ‘implicitly resolved’ in an award; and Judith Prakash J in AQU v AQV [2015] SGHC 262 at [23] that stated “..if, for example, the tribunal's primary finding is that the claimant's claim for breach of contract is inadmissible, the tribunal is not required to address whether or not the respondent's conduct amounted to a breach of the terms of the contract or whether the damages claimed by the claimant were too remote – as referenced in Hill (2018), 'Claims That An Arbitral Tribunal Failed To Deal With An Issue: The Setting Aside Of Awards Under The Arbitration Act 1996 And The UNCITRAL Model Law On International Commercial Arbitration' (2018) 34 Arbitration International, 30.

30 The same was commented by the Working Group - Holtzmann and Neuhaus (1989), “A Guide to the UNCITRAL Model Law on International Commercial Arbitration: Legislative History and Commentary” (Netherlands: Kluwer Law and Taxation Publishers, 1989), 889–907.

31 Ontario (Canada).

32 Such as British Virgin Islands (UK) and Hong Kong (China).

33 Lithuania.

34 Including Austria, Bangladesh, Bhutan, Bulgaria, Denmark, Estonia, Egypt, Germany, Iran, Jordan, Liechtenstein, Lithuania, Malta, Oman, Saudi Arabia, Serbia, Spain, Tunisia, Turkey, UAE, and Uganda.

35 Such as Connecticut (USA), Florida (USA), Illinois (USA), Newfoundland and Labrador (Canada), Northwest Territories (Canada), Nova Scotia (Canada), Nunavut (Canada), Prince Edward Island (Canada), Scotland, Texas (USA), and Zambia.

36 These are specifically Lithuania, Ontario (Canada), and Saskatchewan (Canada).

37 Although it is only mentioned, in the Model Law, that the time limit runs from the date of receipt of the award by the parties (and not each party), it is submitted that if the parties received the award at different dates, then the date of receipt by each party would be the starting date for the time limit of their respective requests.

38 Including Austria (28 days), Bangladesh (14 days), Bhutan (20 days), Bulgaria (60 days), Connecticut (USA – 20 days), Florida (USA – 20 days), Illinois (USA – 20 days), Liechtenstein (28 days), Malta (15 days), Paraguay (15 days), Poland (2 weeks), Qatar (7 days), Saudi Arabia (15 days for correction only), Scotland (28 days), Sri Lanka (14 days), Texas (USA – 20 days), and Uganda (14 days). It should be noted that changing the time limit from 30 days to 28 days (the latter being a multiple of 7) is positive in that it ensures that the deadline does not fall on a weekend.

39 Such as Connecticut (USA), Denmark, Egypt, Florida (USA), Iran, Illinois (USA), Jordan, Maldives, Malta, Oman, Saudi Arabia, Scotland, Serbia, Slovakia, Texas (USA), and Turkey.

40 Such as Poland.

41 Such as Scotland. It should be noted that this is only granted in exceptional cases (see Arbitration Appeal Number 2 of 2019 [2020] CSOH 51 (28 May 2020), where the Scottish Court refused such application) - Leigh Herd, 'Scottish Courts Refuse Application Seeking to Correct or Clarify Arbitrator's Partial Award' (Practical Law UK, 3 June 2020).

42 The phrase ‘of receipt of the request’ indicates the start date of the time limit for the issuance of an article 33 disposition, and even though it is only mentioned for correction and interpretation, it is implied that it also applies to additional award; the omission of this phrase in the sub-article for additional award is thought to be inadvertent - Holtzmann and Neuhaus (1989), “A Guide to the UNCITRAL Model Law on International Commercial Arbitration: Legislative History and Commentary” (Netherlands: Kluwer Law and Taxation Publishers, 1989), 889–907.

43 Such as Poland (2 weeks for correction and interpretation, and 2 months for additional awards), Qatar (7 days), Saudi Arabia (for correction only; 15 days), and Scotland (28 days).

44 Such as Austria, Germany, Jordan (not allowed for correction), Poland, Saudi Arabia (only allowed for additional award), Scotland, Spain, Tunisia (not allowed for correction), and Turkey.

45 Such as Egypt (30 days), Jordan (15 days for interpretation and 30 days for additional award), Oman (30 days), Qatar (7 days), Saudi Arabia (30 days extension for additional award), UAE (15 days extension for correction and interpretation, and 30 days for additional award).

46 The Working Group commented that the requirement for notification was prescribed to allow the non-requesting party opportunity to respond to an article 33 request - Broches (1990), Commentary on the UNCITRAL Model Law on International Commercial Arbitration (Kluwer Law and Taxation Publishers 1990), 171–201.

47 Such as Scotland, Austria, and Spain.

48 It was commented by the Working Group that this requirement flows from the standard of fairness laid out in article 18, which states that ‘[t]he parties shall be treated with equality and each party shall be given a full opportunity of presenting his case.’ - Holtzmann and Neuhaus (1989), “A Guide to the UNCITRAL Model Law on International Commercial Arbitration: Legislative History and Commentary” (Netherlands: Kluwer Law and Taxation Publishers, 1989), 889–907.

49 Such as Germany, Lithuania, Serbia, Slovakia, and Uganda.

50 Such as Belarus, Bulgaria, and Qatar.

51 Under article 51.

52 These include Jordan (unless otherwise agreed), Qatar (unless otherwise agreed), and Belgium.

53 See sub-article 34.4.

54 During the time of the drafting of the Model Law, there was debate as to whether or not to include remission, as it was noted that remission was not a concept known to all legal systems (it was argued that this provision was almost exclusive to common law jurisdictions). However, the Working Group decided on its inclusion as a remedy for a defective award to reduce the chances of an award being set aside - Holtzmann and Neuhaus (1989), “A Guide to the UNCITRAL Model Law on International Commercial Arbitration: Legislative History and Commentary” (Netherlands: Kluwer Law and Taxation Publishers, 1989), 889–907.

55 This is implied from the lack of the contrary ‘agreement of the parties’ phrase usually present in non-mandatory provisions. In addition, article 34 as a whole is mandatory. In fact, during the time of the drafting of the Model Law, one delegation thought that parties to arbitration might not wish to ‘place themselves under the jurisdiction of the court’ and therefore the Model Law should state that the parties could contract out of article 34. Whilst this proposition was not discussed, the fact that it was made and that the legislative history of the Model Law does not indicate that the parties can contract out of article 34 indicate that the parties cannot do so.

56 Including Austria, Bangladesh, Bhutan, Estonia, Egypt, Iran, Japan, Jordan, Liechtenstein, Oman, Saudi Arabia, Slovakia, South Korea, Spain, and Turkey.

57 See, e.g., Qatar.

58 Such as Germany and Scotland.

59 Including Georgia (not more than 30 days) Hungary (not more than 90 days), and UAE (not more than 60 days).

60 Lack of clarity regarding this issue was commented by multiple delegates during the time of the drafting of the Model Law. See, International commercial arbitration - Travaux préparatoires at pages 249–253 of A/CN.9/246, annex; A/CN.9/263 and Add.1-2; A/CN.9/264; & at pages 454–458 of A/CN.9/246, annex; A/CN.9/263 and Add.1-2; A/CN.9/264; A/CN.9/XVIII/CRP.1 and 3–6; UNCITRAL Model Law on International Commercial Arbitration (1985) – Link: https://uncitral.un.org/sites/uncitral.un.org/files/media-documents/uncitral/en/318meeting-e.pdf & https://uncitral.un.org/sites/uncitral.un.org/files/media-documents/uncitral/en/319meeting-e.pdf.

61 See sub-article 54(6).

62 Such as changes to default time limits for issuance of article 33 dispositions, when these dates may be extended by the arbitral tribunal.

63 Such as changes to the interpretation provision; as interpretation is rarely requested for and even more rarely assented to in international arbitration.

64 Such as changes to the availability and mandatory characteristic of the provisions of article 33 and sub-article 34.4.

65 Such as changes to the ability of the arbitral tribunal to extend the time limit for the issuance of an article 33 disposition, which may cause an article 33 disposition to be impractical or result in such dispositions being void for breach of natural justice; mostly notably in case of additional awards.

66 Such as allowing for interpretation and additional award on the arbitral tribunal's own initiative.
